# Identification of Genes Involved in Low Temperature-Induced Senescence of Panicle Leaf in *Litchi chinensis*

**DOI:** 10.3390/genes10020111

**Published:** 2019-02-01

**Authors:** Congcong Wang, Hao Liu, Sheng Yu, Houbin Chen, Fuchu Hu, Huiling Zhan, Xifen Pan, Yuhua Lao, Silin Zhong, Biyan Zhou

**Affiliations:** 1 Guangdong Litchi Engineering Research Center, College of Horticulture, South China Agricultural University, Guangzhou 510642, China; 15240667015@163.com (C.W.); maomihaoge@163.com (H.L.); hbchen@scau.edu.cn (H.C.); 13760897188@163.com (H.Z.); 13760897403@163.com (X.P.); low0517@163.com (Y.L.); 2 Guangdong Academy of Forestry, Guangzhou 510520, China; 3 State Key Laboratory of Agrobiotechnology, School of Life Sciences, Chinese University of Hong Kong, Hong Kong, China; yusheng@link.cuhk.edu.hk; 4 Key Laboratory of Tropic Fruit Trees, Hainan Academy of Agricultural Science, Haikou 571100, China; hufuchu@163.com

**Keywords:** panicle leaf, senescence, transcriptome, litchi, low temperature

## Abstract

Warm winters and hot springs may promote panicle leaf growing and repress floral development. To identify genes potentially involved in litchi panicle leaf senescence, eight RNA-sequencing (RNA-Seq) libraries of the senescing panicle leaves under low temperature (LT) conditions and the developing panicle leaves under high temperature (HT) conditions were constructed. For each library, 4.78–8.99 × 10^6^ clean reads were generated. Digital expression of the genes was compared between the senescing and developing panicle leaves. A total of 6477 upregulated differentially expressed genes (DEGs) (from developing leaves to senescing leaves), and 6318 downregulated DEGs were identified, 158 abscisic acid (ABA)-, 68 ethylene-, 107 indole-3-acetic acid (IAA)-, 27 gibberellic acid (GA)-, 68 cytokinin (CTK)-, 37 salicylic acid (SA)-, and 23 brassinolide (BR)-related DEGs. Confirmation of the RNA-Seq data by quantitative real-time PCR (qRT-PCR) analysis suggested that expression trends of the 10 candidate genes using qRT-PCR were similar to those revealed by RNA-Seq, and a significantly positive correlation between the obtained data from qRT-PCR and RNA-Seq were found, indicating the reliability of our RNA-Seq data. The present studies provided potential genes for the future molecular breeding of new cultivars that can induce panicle leaf senescence and reduce floral abortion under warm climates.

## 1. Introduction

Litchi is an evergreen fruit tree widely cultivated in Southeast Asia. Flowering can be regarded as the transformation from vegetative growth to reproductive growth. It has been demonstrated that low temperature is an indispensable factor for litchi flowering [1,2]. A cool winter confirms reproductive growth for this evergreen fruit tree [3], whereas a warm winter may disrupt the reproductive growth and promote vegetative growth. Hence, the competition between vegetative growth and reproductive growth normally exists in winter and early spring, during which litchi is undergoing floral induction and differentiation.

Litchi floral buds are mixed buds with both the panicle primordia as the reproductive structure and rudimentary leaves (panicle leaves) as the potential vegetative structure. When field temperature is low enough, the panicle primordia can develop, while the rudimentary leaves may stop developing and senesce before expanding. When warm winters and hot springs attributed to global warming happen, the rudimentary leaves may elongate and expand quickly. The vegetative growth prevails and the reproductive structure ceases developing, resulting in poor flowering [4]. How to induce senescence or abscission of the panicle leaves is of great importance for flowering regulation in litchi. The most effective way is to provide a low enough temperature condition for floral induction and differentiation. However, due to climate change and global warming, litchi trees may be exposed to high temperature conditions during these critical periods. Chemicals, such as the ethylene releaser ethephon, the reactive oxygen species (ROS) inducer methyl viologen dichloride hydrate (MV), and the nitric oxide (NO) donor sodium nitroprusside (SNP), may be used in place of low temperatures to promote the panicle leaf senescence and abscission [5].

Leaf senescence is the final stage of leaf development [6,7]. The process may occur automatically in mature leaves as age-dependent senescence [8]. Also, leaves may be induced to senesce before maturity when they are exposed to drought, and high and low temperatures [9,10,11]. The litchi panicle leaf senescence induced by low temperature, ROS, and NO is regarded as premature leaf senescence [3,12,13]. In our previous studies, we have figured out the feature of the low temperature-induced senescence of panicle leaves, and found that programmed cell death (PCD) is involved in the senescence induced by low temperatures in litchi [3,12]. We then determine the expression profiles of PCD-related genes during the low temperature-induced senescence [12]. However, the expression patterns of other genes during low temperature-induced senescence in litchi panicle leaves are not known. It has been demonstrated that leaf senescence is controlled by hormonal signaling regulatory gene networks. Abscisic acid (ABA), ethylene, jasmonic acid (JA), and salicylic acid (SA) signaling pathways can speed up senescence in older leaves [14,15], while cytokinin (CTK), gibberellic acid (GA), and auxin (IAA) delay the onset of senescence [8]. It has also been shown that transcription factors NAC, WRKY, and MYB are central players in modulating transcriptional changes under hormonal control [16,17]. Understanding how low temperature affects hormone-related gene expression and their central players is the critical clue for panicle leaf control.

RNA-sequencing (RNA-Seq) technology is a powerful tool for plant senescence studies. It has been used to identify genes involved in MV-generated ROS-induced senescence of rudimentary leaves in litchi [18], MADS-box family genes associated with stress resistance in *Brassica rapa* [19], and compare transcriptomes between low- and high-cadmium-accumulating *Brassica chinensis* genotypes in response to cadmium stress [20]. In the present study, we used RNA-Seq technology to identify hormone-related DEGs, compare the transcriptome of the low temperature-induced senescent panicle leaves with those of the developing ones under normal temperature conditions. We aim to reveal the molecular mechanism of the panicle leaf senescence, and to provide potential genes for genetic control of panicle leaf growth to produce high quality flowers under global warming conditions.

## 2. Materials and Methods

### 2.1. Plant Materials

Four-year-old ‘Nuomici’ litchi trees (*Litchi chinensis*) were used in this study. The trees were cultivated in the experimental orchard of South China Agricultural University, Guangzhou, China (lat. 23°9′40″ N, long. 113°21′18″ E). All trees were planted in 30 L pots which contained loam, mushroom cinder, and coconut chaff (3:1:1 (*v*/*v*/*v*)). Trees with leaves of the terminal shoots at the same maturity stage were selected. The trees were subjected to winter chilling for floral induction in open field. When panicle primordia were visible as ‘whitish millets’, 10 potted trees were moved to growth chambers at 18 °C as low temperature (LT) treatment to induce panicle leaf senescence and panicle development. Another 10 potted trees were transferred to 26 °C chambers to promote panicle leaf senescence and panicle development. Three replicated samples of developing leaves at an early stage (DLE), and those of the developing leaves at a later stage (DLL) under high temperature (HT) were collected from trees under HT condition. Also, senescing leaves at an early stage (SLE) and senescing leaves at a later stage (SLL) were collected from trees grown in LT condition. All samples were frozen in nitrogen immediately and stored at −80 °C for RNA extraction.

### 2.2. RNA Isolation, cDNA Synthesis, and Library Construction for Transcriptome

Total RNA of DLE, DLL, SLE, and SLL were isolated from leaf tissues according to the Plant Total RNA Isolation Kit manual (Huayueyang, Beijing, China). The quantity and purity of total RNA were measured by ultraviolet spectrophotometer (Eppendorf, Hamburg, Germany) and the samples with 260/280 nm ratio between 1.8 and 2.0 were selected for subsequent complementary DNA (cDNA) synthesis. SuperScript III (Invitrogen, Carlsbad, CA, USA) and primer oligo d(T)18 were used to synthesize the first-strand cDNA for the subsequent experiment. The messenger RNA (mRNA) from total RNA was enriched using Oligo-dT beads (Qiagen, Valencia, CA, USA) and fragmented into short fragments using fragmentation buffer. The short mRNA fragments were reversed transcribed into cDNA by random primers and second-strand cDNA synthesized using DNA polymerase I, RNase H, and dNTPs. QIAquick PCR Purification Kit (Qiagen) was used to purify cDNA fragments. Then, poly(A) tails were added to the repaired purified cDNA fragments, followed by ligation to Illumina sequencing adapters. After PCR amplification, two biological replicates of DLE, DLL, SLE, and SLL library preparations were sequenced on an Illumina HiSeq^TM^ 2500 platform at BGI (Shenzhen, China) using a 50 single-end (SE) module. 

### 2.3. RNA-Seq Data Analysis and Gene Functional Annotation

The raw reads of transcriptome were pre-processed by removing adaptor sequences and low-quality sequences. Filtered reads were mapped to the litchi reference genome using Bowtie2 (v2.1.0., [21]). Expression levels of each unigene were quantified using fragments per kilobase of transcript per million mapped reads (FPKM). The read counts and FPKM calculation of each transcript were computed with the parameter settings followed by the manual of eXpress (v1.5.1., [22]). The reads were available at the National Center for Biotechnology Information Short Read Archive (SRA) by the accession number PRJNA430479 [23]. Differential expression analysis was detected by DESeq2 [24]. Differentially expressed genes (DEGs) were screened using false discovery rate (FDR) ≤0.01. Gene ontology (GO) terms for the litchi transcripts were obtained from a Blastp search against the UniProtKB Swiss-Prot database [25]. The enrichment of the GO functional classifications was plotted using the R Bioconductor package GOstats [26].

### 2.4. Quantitative Real-Time PCR Analysis

To validate the digital gene expression data, quantitative real-time PCR (qRT-PCR) was used. First-strand cDNA was synthesized using M-MLV reverse transcriptase (Invitrogen). The cDNA was used as templates in qRT-PCR according to the SYBR Premix Ex Taq manual (TaKaRa Biotechnology, Dalian, China) on a LightCycler 480 real-time PCR machine (Roche, Basel, Switzerland). The primers were designed by Primer 6.0 (Premier Biosoft, Palo Alto, CA, USA) and listed in Table S1. The protocol with qRT-PCR and reference genes was performed according to Lu et al. [18]. The relative expression level of candidate genes was calculated by 2^−^^∆∆CT^ method [27] with three biological and three technical replicates.

### 2.5. Statistical Analysis

Data were analyzed by SPSS (version 19.0; IBM Corp., Armonk, NY, USA) and expressed as mean ± standard error (SE)) using Origin (version 9.1; OriginLab Corp., Northampton, MA, USA). Significant differences among treatments and controls were determined by Tukey test at 0.05 and 0.01 probability levels. 

## 3. Results

### 3.1. Morphology of Senescing and Developing Panicle Leaves

**Figure 1:**
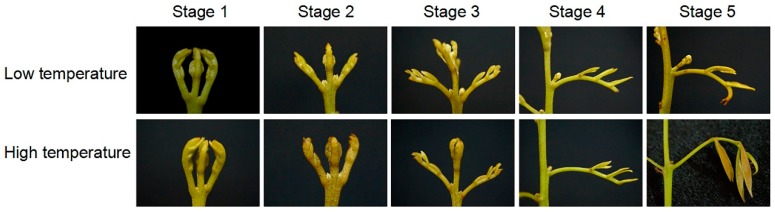
The phenotype of five developmental stages of panicle leaves in litchi trees subjected to low or high temperatures. Litchi trees were transferred to a growth chamber at 18 °C as low temperature treatment to induce panicle leaf senescence when panicle primordia emerged. The other trees were transferred to a growth chamber at 26 °C as high temperature to encourage panicle leaf development. The growth of the panicle leaves was divided into 5 stages.

To identify genes involved in low temperature-induced panicle leaf senescence, we transferred the potted trees to LT growth chambers to induce panicle leaf senescence, or HT growth chambers to allow panicle leaf development as control trees. Figure 1 shows the morphology of the senescing and developing panicle leaves during the LT or HT treatments. Under LT condition, at stage 1 and 2, the primary stages of senescence, the leaflets of the compound leaves were conglutinated. At stage 3, as the middle stage of senescence, the leaves began to elongate. At stage 4, the petiole of the rudimentary leaf began to elongate. At this stage, each leaflet could be identified. At stage 5, the petiole of the rudimentary leaf ceased expanding. During these 5 stages, the panicle primordia developed. At stage 1 to stage 4 under HT condition, panicle leaves showed similar morphological changes as those under LT condition. However, at stage 5, the panicle leaves started to expand. During the 5 stages at HT, the panicle primordia ceased to develop and shrank.

### 3.2. Digital Transcriptome Analysis

To identify genes potentially involved in LT-induced panicle leaf senescence, we collected panicle leaves at stage 1 at LT as the early senescent tissues (SLE), and at stage 3 as later senescent tissue (SLL). Those at HT as the DLE and DLL were controls. We constructed eight RNA-Seq libraries for SLE, SLL, DLE, and DLL. As shown in Table 1, we have generated 4.83–9.08 × 10^6^ raw reads and obtained 4.78–8.99 × 10^6^ clean reads from the libraries, with clean read ratios of more than 95%. We then mapped the clean reads to the litchi transcriptome and obtained at least 55% mapping rate. The mapped reads for the six libraries ranged from 3.21 × 10^6^ to 5.48 × 10^6^.

### 3.3. DEG Identification and GO-Term Analysis

**Figure 2:**
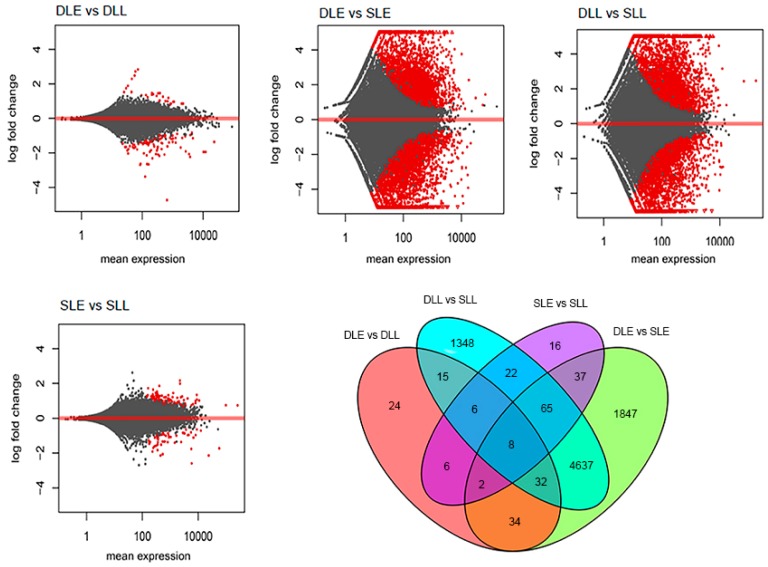
Analysis of the differentially expressed genes between samples. X-axis indicates the mean expression of the compared genes. Y-axis indicates the log2 fold of the compared genes. Red dots indicate differentially expressed genes. Gray dots indicate non-differentially expressed genes.

We remapped the unique match reads from these eight libraries to the reference sequences and normalized the unigene reads to FPKM values. Correlation analysis of the FPKM values for the replicates of DLE, DLL, SLE, and SLL showed high repeatability of the sequencing samples (Figure S1). We then conducted pair comparison to identify DEGs using DESeq2 and identified 13,084 DEGs. As shown in Figure 2 and Table 2, most of the DEGs were from the comparison of SLE and DLE, or SLL and SLL, whereas only 127 were from the comparison of SLE and SLL, 162 from that of DLE and DLL. The results suggested that almost all the genes at stage 1 and 3 of the senescing or developing panicle leaves were at the same expression levels, while the expression levels between the senescing panicle leaves and the developing ones were quite different. The DEGs between developing leaves and senescing leaves at the early stage were 6662, and those at the later stage were 6133. We identified 6477 upregulated DEGs (from developing leaves to senescing leaves), and 6318 downregulated DEGs. GO-term analysis was performed for all the DEGs. The enriched GO-terms are shown in Figure S1. They were classified into the cellular component, biological process, and molecular function. The DEGs between senescing panicle leaves and developing ones were the combination of those between SLE and DLE, and between SLL and DLL.

### 3.4. Identification of the Plant Hormone-Related Genes Potentially Involved in LT-Induced Panicle Leaf Senescence

**Figure 3:**
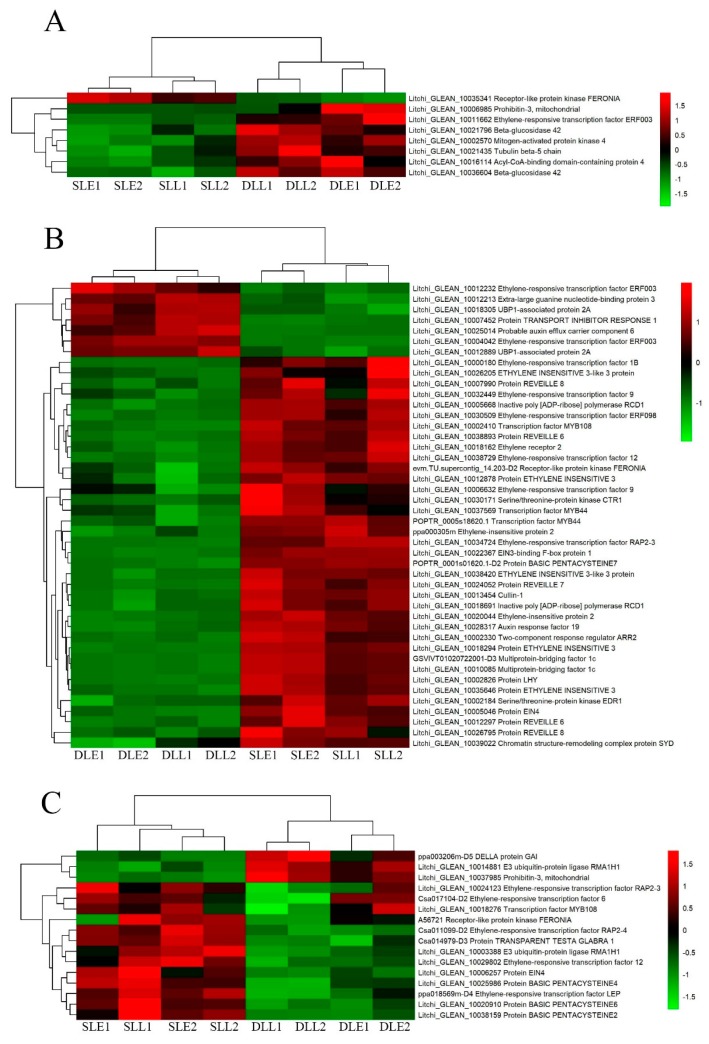
Heat map diagram showing the ethylene-related differentially expressed genes (DEGs) between senescing and developing panicle leaves. (**A**) The unique DEGs expressed at an early stage. (**B**) The DEGs simultaneously expressed at early and later stages. (**C**) The unique DEGs expressed at a later stage. Litchi trees were transferred to a growth chamber at 18 °C to induce panicle leaf senescence when panicle primordia emerged. The other trees were transferred to a growth chamber at 26 °C to encourage panicle leaf development. Fragments per kilobase of transcript per million mapped read (FPKM) values of the samples were normalized to Z-score.

**Figure 4:**
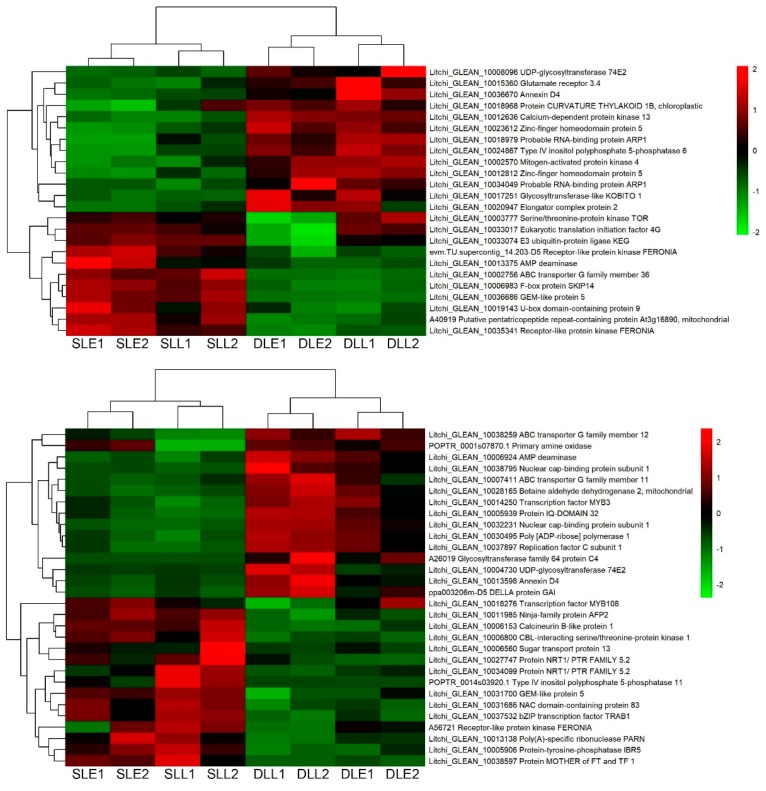
Heat map diagram showing the unique ABA-related differentially expressed genes (DEGs) between senescing and developing panicle leaves at an early stage (upper panel) and those at a later stage (lower panel). Litchi trees were transferred to a growth chamber at 18 °C to induce panicle leaf senescence when panicle primordia emerged. The other trees were transferred to a growth chamber at 26 °C to encourage panicle leaf development. FPKM values of the samples were normalized to Z-score.

**Figure 5:**
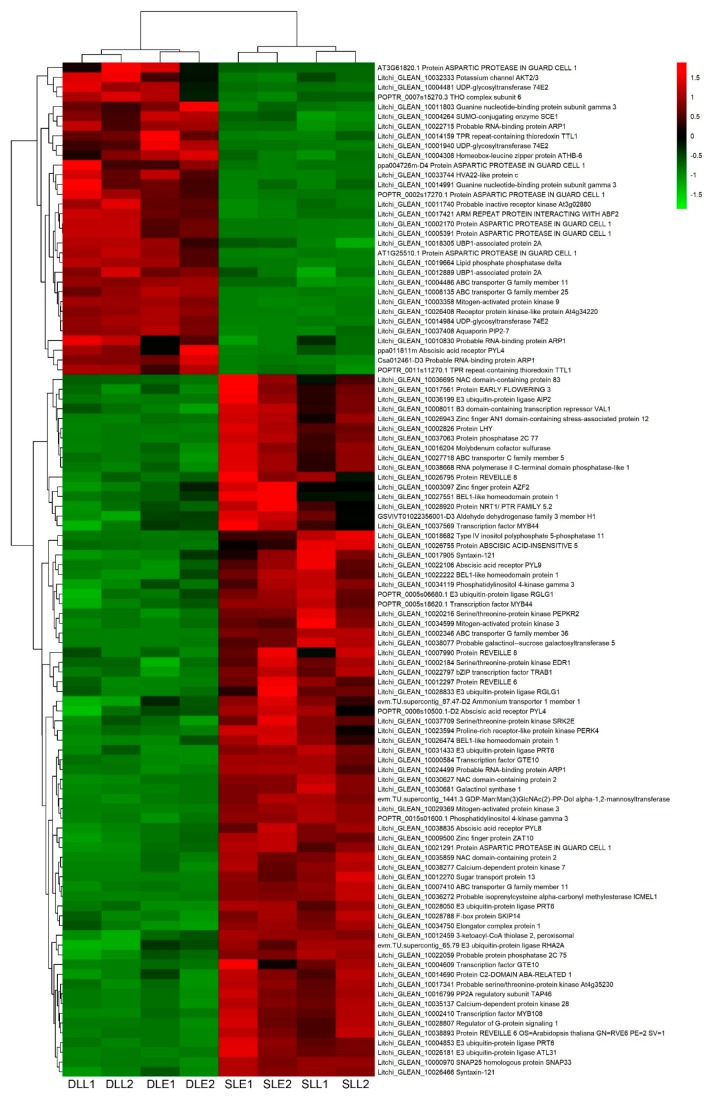
Heat map diagram showing the ABA-related differentially expressed genes (DEGs) between senescing and developing panicle leaves simultaneously expressed at early and later stages. Litchi trees were transferred to a growth chamber at 18 °C to induce panicle leaf senescence when panicle primordia emerged. The other trees were transferred to a growth chamber at 26 °C to encourage panicle leaf development. FPKM values of the samples were normalized to Z-score.

**Figure 6:**
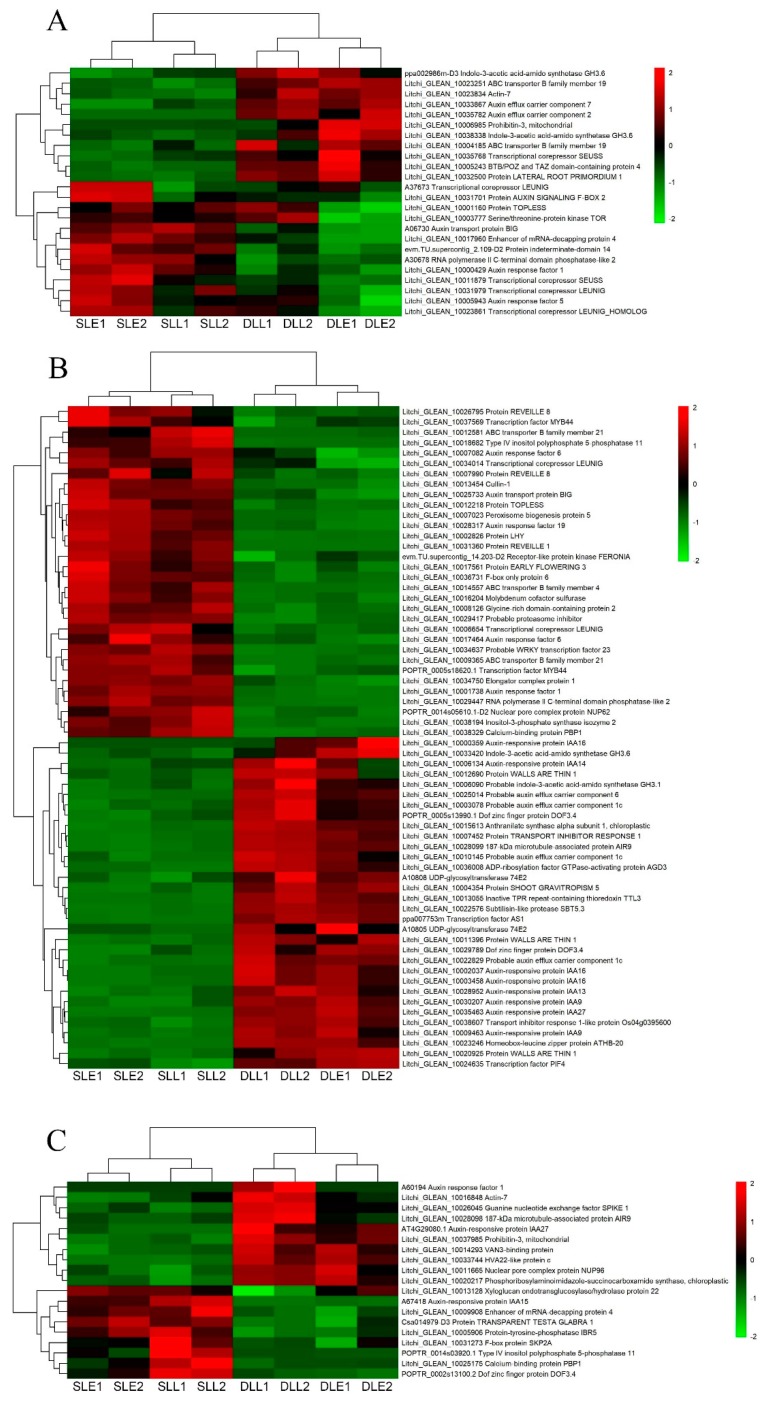
Heat map diagram showing the IAA-related differentially expressed genes (DEGs) between senescing and developing panicle leaves. (**A**) The unique DEGs expressed at an early stage. (**B**) The DEGs simultaneously expressed at early and later stages. (**C**) The unique DEGs expressed at a later stage. Litchi trees were transferred to a growth chamber at 18 °C to induce panicle leaf senescence when panicle primordia emerged. The other trees were transferred to a growth chamber at 26 °C to encourage panicle leaf development. FPKM values of the samples were normalized to Z-score.

**Figure 7:**
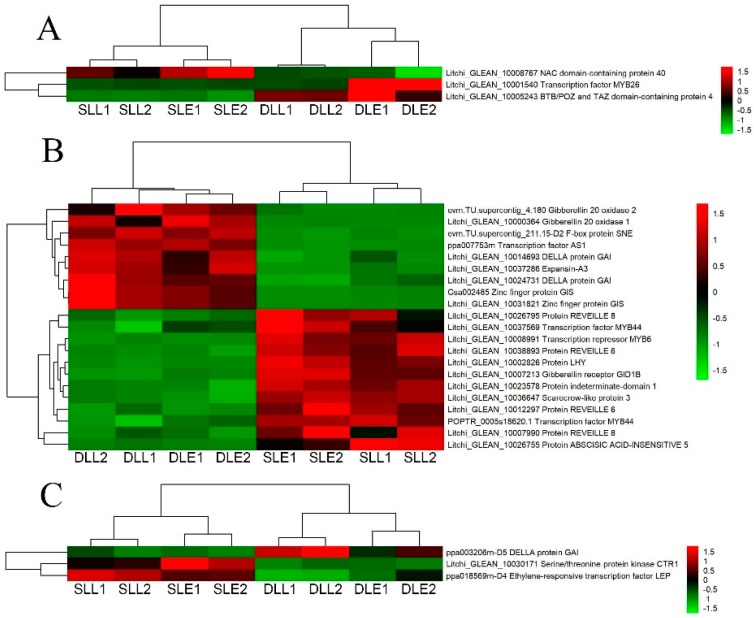
Heat map diagram showing the GA-related differentially expressed genes (DEGs) between senescing and developing panicle leaves. (**A**) The unique DEGs expressed at an early stage. (**B**) The DEGs simultaneously expressed at early and later stages. (**C**) The unique DEGs expressed at a later stage. Litchi trees were transferred to a growth chamber at 18 °C to induce panicle leaf senescence when panicle primordia emerged. The other trees were transferred to a growth chamber at 26 °C to encourage panicle leaf development. FPKM values of the samples were normalized to Z-score.

**Figure 8:**
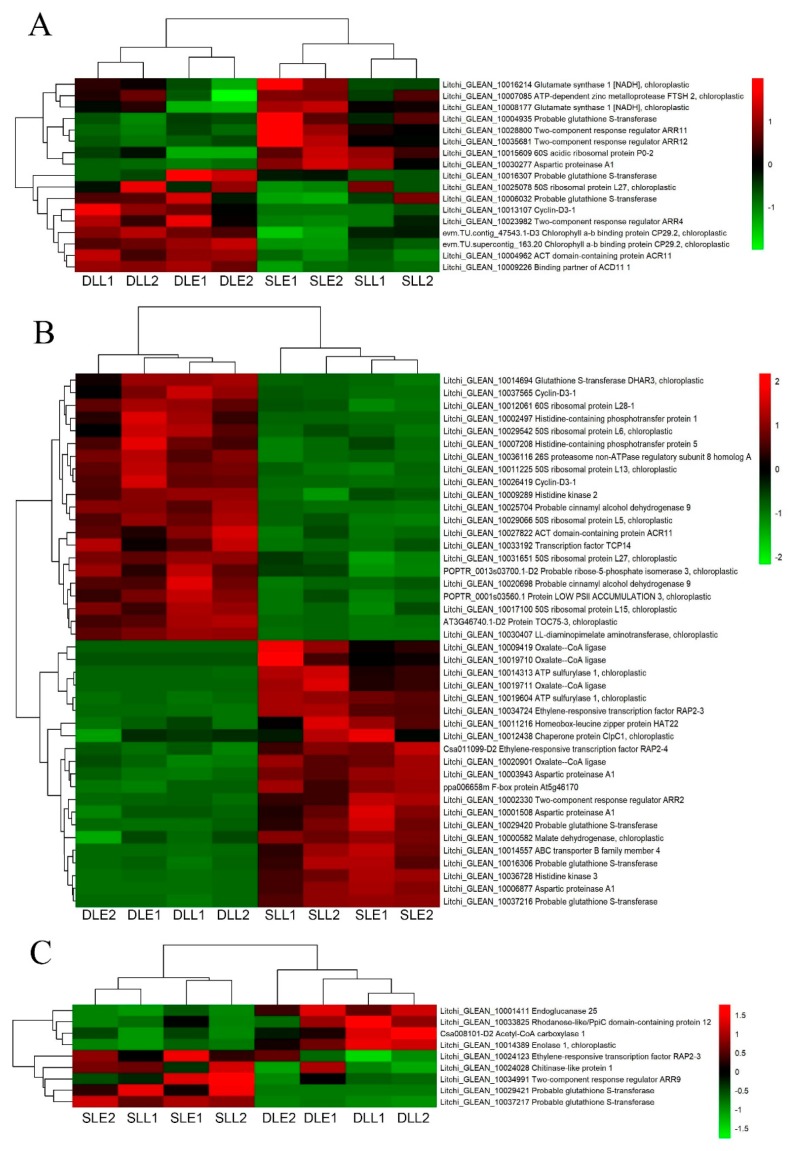
Heat map diagram showing the CTK-related differentially expressed genes (DEGs) between senescing and developing panicle leaves. (**A**) The unique DEGs expressed at an early stage. (**B**) The DEGs simultaneously expressed at early and later stages. (**C**) The unique DEGs expressed at a later stage. Litchi trees were transferred to a growth chamber at 18 °C to induce panicle leaf senescence when panicle primordia emerged. The other trees were transferred to a growth chamber at 26 °C to encourage panicle leaf development. FPKM values of the samples were normalized to Z-score

**Figure 9:**
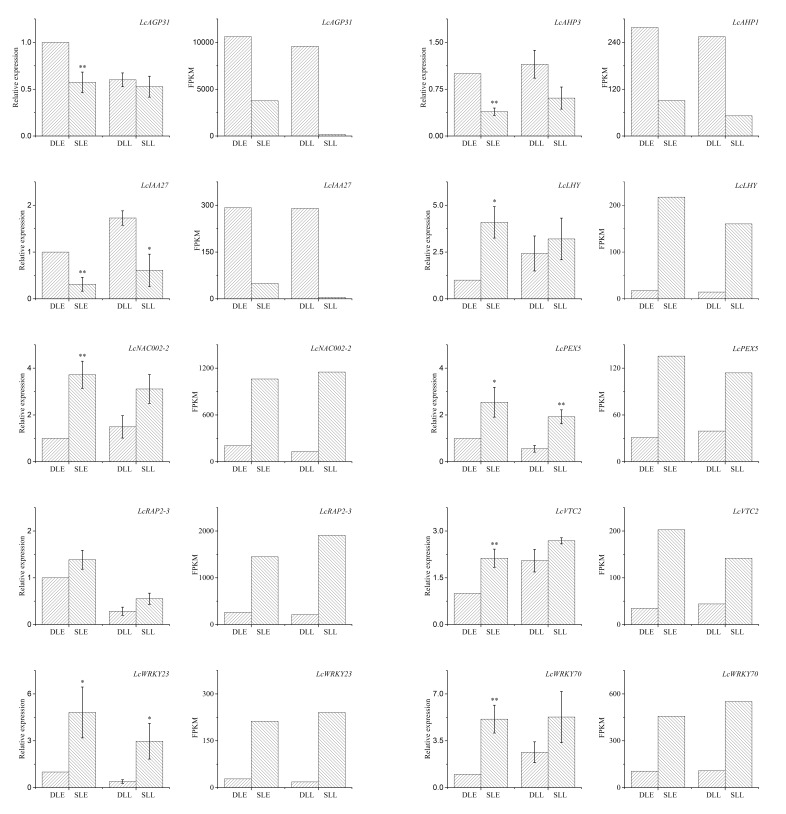
Expression of the 10 candidate differentially expressed genes determined by quantitative real-time PCR (qRT-PCR) and RNA-sequencing (RNA-Seq). Single asterisk indicates significant difference between developing leaves and senescing leaves at 0.05 probability level. Double asterisks indicate significant difference between developing leaves and senescing leaves at 0.01 probability level.

From the enriched GO-terms related to plant hormones, we identified 68 ethylene-, 158 abscisic acid (ABA)-, 107 indole-3-acetic acid (IAA)-, 68 cytokinin (CTK)-, 37 salicylic acid (SA)-, 66 jasmonic acid (JA)-, and 23 brassinolide (BR)-related DEGs that might be involved in the LT-induced panicle leaf senescence (Figure 3, Figure 4, Figure 5, Figure 6, Figure 7, Figure 8 and Figure 9). These GO-terms were response to hormone, hormone-mediated signaling pathway, ethylene-activated signaling pathway, jasmonic acid-mediated signaling pathway, cellular response to hormone stimulus, cellular response to ethylene stimulus, response to cytokinin, auxin efflux transmembrane transporter activity, auxin transmembrane transporter activity, regulation of hormone metabolic process, salicylic acid metabolic process, regulation of salicylic acid metabolic process, and hormone activity.

Figure 3 shows the ethylene-related DEGs. Eight genes at an early stage and 3 genes at a later stage were only differentially expressed between the senescing and developing panicle leaves. Twenty-one DEGs were simultaneously expressed at the two stages. Interestingly, from developing leaves to sensing leaves, 51 out of 68 of the DEGs were upregulated, such as the ethylene receptor 2, protein EIN4, ethylene-responsive transcription factor 1B, ethylene-responsive transcription factor 9, ethylene-responsive transcription factor RAP2-3, and MYB44 encoding genes. 

Twenty-four genes at an early stage and 30 genes at a later stage were only differentially expressed between the senescing and developing panicle leaves (Figure 4). One hundred and four DEGs were simultaneously expressed at the two stages (Figure 5). Interestingly, most of the DEGs from developing leaves to sensing leaves showed upregulated trends. Ninety-eight out of 158 DEGs were upregulated, such as the ABA receptor PYL8, ABA receptor PYL9, protein phosphatase 2C 77, serine/threonine-protein kinase SRK2E, protein LHY, NAC domain-containing protein 83, NAC domain-containing protein 2, and transcription factor MYB44 encoding genes. 

Figure 6 shows the IAA-related DEGs. Twenty-four genes at the early stage and 19 genes at the later stage were only differentially expressed between the senescing and developing panicle leaves (Figure 6A,C). Sixty-four DEGs were simultaneously expressed at the two stages (Figure 6B). From the developing leaves to sensing leaves, about half of the DEGs were downregulated, such as the probable auxin efflux carrier component 6, probable auxin efflux carrier component 1C, auxin-responsive protein IAA9, auxin-responsive protein IAA13, auxin-responsive protein IAA14, auxin-responsive protein IAA16, and auxin-responsive protein IAA27 encoding genes. Another half of the DEGs were upregulated, such as the auxin response factor 6, auxin transport protein BIG, protein EARLY FLOWERING 3, probable WRKY transcription factor 23, and transcription factor MYB44 encoding genes.

As shown in Figure 7, only 27 GA-related DEGs were identified. Three genes at the early stage and 3 genes at the later stage were only differentially expressed between the senescing and developing panicle leaves (Figure 7A,C). Twenty-one DEGs were simultaneously expressed at the two stages. Among the GA-related DEGs, 15 DEGs showed upregulated trends from the developing panicle leaves to the senescing ones, such as the gibberellin receptor GID1B12, transcription factor MYB44, transcription repressor MYB6, protein LHY, and NAC domain-containing protein 40 encoding genes. Twelve DEGs showed downregulated trends, such as the DELLA protein GAI, gibberellin 20 oxidase 1, gibberellin 20 oxidase 2, F-box protein SNE, and zinc finger protein GIS encoding genes. 

Figure 8 shows the CTK-related DEGs. Seventeen genes at an early stage and 9 genes at a later stage were only differentially expressed between the senescing and developing panicle leaves. Forty-two DEGs were simultaneously expressed in the two stages. From the developing leaves to sensing leaves, half of the DEGs were upregulated, such as the two-component response regulator ARR2, two-component response regulator ARR11, two-component response regulator ARR12, oxalate-CoA ligase, and probable glutathione *S*-transferase encoding genes. Another half of the DEGs were downregulated, such as the two-component response regulator ARR4, chlorophyll a/b binding protein CP29.2, ATC domain-containing protein ACR11, and acetyl-CoA carboxylase encoding genes. 

Figure S3 shows the JA-related DEGs. Fifteen genes at an early stage and 10 genes at a later stage were only differentially expressed between the senescing and developing panicle leaves. Forty-one DEGs were simultaneously expressed at the two stages. From the developing leaves to sensing leaves, 44 DEGs were upregulated, such as the regulatory protein NPR3, probable WRKY transcription factor 51, probable WRKY transcription factor 70, transcription factor MYB 6, and transcription factor MYB 108 encoding genes. Twenty-two of the DEGs were downregulated, such as the transcription factor MYB 108, protein NRT1/PTR FAMILY 5.2, the BTB/POZ, and TAZ domain-containing protein 4 encoding genes. 

Figure S4 shows the SA-related DEGs. Five genes at an early stage and 9 genes at a later stage were only differentially expressed between the senescing and developing panicle leaves. Twenty-three DEGs were simultaneously expressed in the two stages. From the developing leaves to sensing leaves, 28 DEGs were upregulated, such as the serine/threonine-protein kinase EDR1, and SUMO-protein ligase SIZ1 encoding genes. Nine DEGs were downregulated, such as the Dof zinc finger protein DOF3.4, transcription factor AS1, and HAV22-like protein C encoding genes. 

Figure S5 shows the BR-related DEGs. Four genes at an early stage and 7 genes at a later stage were only differentially expressed between the senescing and developing panicle leaves. Twelve DEGs were simultaneously expressed in the two stages. From the developing leaves to sensing leaves, 11 DEGs were upregulated, such as the serine/threonine-protein kinase TOR, receptor-like protein kinase FERONIA, and transcription factor bHLH75 encoding genes. Twelve DEGs were downregulated, such as the BRASSINOSTEROID INTENSIVE 1, lysine-specific demethylaseREF6, and inactive TPR repeat-containing thioredoxin TTL3 encoding genes. 

### 3.5. Validation of the Expression Pattern of DEGs by qRT-PCR

**Figure 10:**
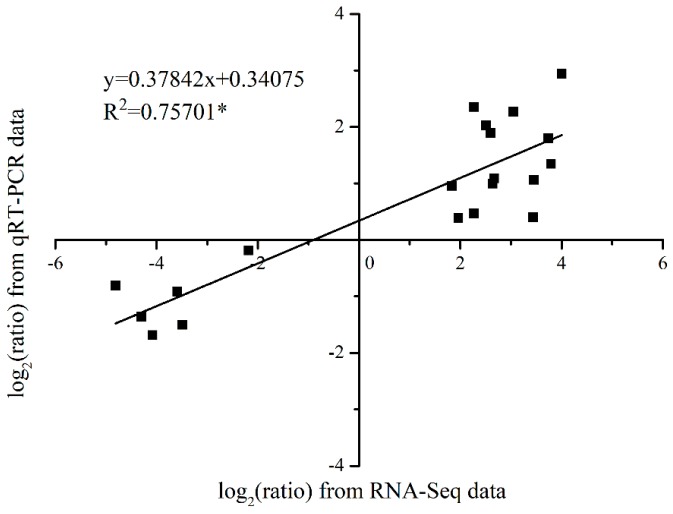
Coefficient analysis of fold changes data between qRT-PCR and RNA-Seq. Scatter plots represent the fold-changes in the gene expression levels of the senescing panicle leaves compared to those of the developing panicle leaves. All the data are from the qRT-PCR analysis and RNA-Seq of the DLE, DLL, SLE, and SLL. Asterisk indicates that the slope is significantly different from zero at 0.05 probability level.

To confirm the RNA-Seq data, we selected 10 plant hormone-related DEGs and determined their relative expression using qRT-PCR. As shown in Figure 9, from the developing panicle leaves to the senescing ones at the two developmental stages, expression of *LcAGP31*, *LcAHP1*, and *LcIAA27* showed decreasing trends, while *LcLHY*, *LcNAC002-2*, *LcPEX5*, *LcRAP2-3*, *LcVTC2*, *LcWRKY23*, and *LcWRKY70* showed increasing trends. The results showed that their expression trends as determined by qRT-PCR were similar to those revealed by RNA-Seq, suggesting that the RNA-Seq data are reliable (Figure 9). Moreover, a linear regression analysis results of the fold-change in the gene expression ratios between RNA-Seq and qRT-PCR showed a significantly positive correlation between the obtained data from qRT-PCR and RNA-Seq, and further confirmed the reliability of our RNA-Seq data (Figure 10).

## 4. Discussion

Leaf senescence is the final stages of growth and development [7]. This process is always related to the yield of field crops. Under suitable growth conditions, leaf senescence starts in an age-dependent manner [8]. Delaying this age-dependent senescence can increase crop yield. There is another type of leaf senescence, the premature senescence induced by drought, and high or low temperature stresses [9,10,11]. Crop growers try to avoid premature leaf senescence. Interestingly, litchi panicle leaves can be regarded as the vegetative structures that may hamper floral development. Litchi growers always inhibit the development or induce premature senescence of these panicle leaves to guarantee reproductive growth, in other words, to increase litchi fruit yield [3,4,5]. Hence, this cultivation practice for litchi is different from that for field crops. During litchi panicle development, appropriate low temperatures can induce premature senescence of panicle leaves. 

In the present study, we performed genome-wide transcriptome analysis of the litchi panicle leaves of the potted trees under low temperature condition as treatment, and those under high temperature condition as a control. We constructed eight RNA-Seq libraries, generating 4.78–8.99 × 10^6^ clean reads for each library. To identify genes potentially involved in low temperature-induced panicle leaf senescence, we compared digital expression of the genes in the SLE with that in the DLE, and in the SLL with that in the DLL. As a result, we identified 6477 upregulated DEGs (from developing leaves to senescing leaves) and 6318 downregulated DEGs. Then, GO-term analysis was performed among the DEGs between the developing leaves and senescing leaves. We found that the hormone-related terms were significantly enriched. Mainly basing on these terms, we identified ABA-, ethylene-, IAA-, GA-, CTK-, SA-, and BR-related DEGs that might be involved in panicle leaf senescence. 

Plant hormones are signal molecules produced in plant cells which are expressed at extremely low concentrations but can regulate plant growth and development [28]. Ethylene is regarded as a promoter for abscission, which is the final stage of senescence. Deficiency in the ethylene signaling or biosynthesis genes affects senescence [29]. Exogenous application of ethylene induces panicle leaf senescence in litchi [5]. ABA is an essential hormone to promote senescence. Increased ABA levels are found in senescent leaves [3,30]. Interestingly, from developing leaves to senescing leaves, 51 out of 68 of the ethylene-related DEGs and 90 out of 158 ABA-related DEGs were upregulated, such as the ethylene receptor 2, protein EIN4, ethylene-responsive transcription factor 1B, the ABA receptors PYL8 and PYL9, and protein phosphatase 2C 77. These results indicated that many more ethylene- and ABA-related genes were involved in the promotion of the LT-induced panicle leaf senescence. In accordance with the present studies, our previous studies showed that ethylene and ABA treatments could induce panicle leaf senescence and abscission, and promote panicle development [5,31]. Interestingly, apart from LT, ROS also induce litchi panicle leaf senescence [4,13]. It was found that genes encoding signaling components of ethylene and ABA were significantly enriched during the ROS-induced senescence processes [8], suggesting that the ethylene- and ABA-related genes may play important roles in the regulation of senescence. Ethylene and ABA may be widely involved in the promotion of senescence and abscission. 

IAA and GA, in general, are regarded as the senescence-retarding hormones. Exogenous application of IAA can repress the expression of the senescence-associated gene *SAG12* which is regarded as the molecular marker of senescence, and delay senescence [8]. GA can delay the senescence of individual leaves [8]. Cytokinins modulate senescence, which is associated with chlorophyll breakdown, photosynthetic apparatus disintegration, and oxidative damage [32]. Exogenous application of CTK delayed flag leaf senescence of wheat [33]. Our present study showed that from the developing leaves to senescing leaves, the number of the downregulated DEGs in relation to IAA, GA, and CTK was similar to that of the upregulated DEGs, and was different from those in relation to ethylene and ABA, suggesting that IAA, GA, and CTK played different roles in LT-induced panicle leaf senescence. 

JA is a crucial hormone that modulates multiple physiological processes, such as anthocyanin accumulation, trichome initiation, leaf senescence, and biotic and abiotic stress responses [34]. JA induces leaf senescence in plant species and, hence, is regarded to be a promoter of leaf senescence [35]. SA is also considered to be an inducer of leaf senescence. Increased SA levels are detected in senescent leaves [36]. Brassinosteroids, which are structurally similar to steroid hormones in animals, impact the activity of numerous metabolic pathways and help control the growth and development in relation to morphogenesis [37]. Exogenous brassinosteroid application delays senescence in papaya leaves [38]. In this study, we identified many JA-, SA-, and BR-related DEGs. Intriguingly, compared to the downregulated (from developing leaves to sensing leaves) DEGs, we found many more upregulated DEGs in relation to the senescence promoter JA and the inducer SA. As for BR, the number of the upregulated DEGs are similar to that of the downregulated ones. It seems that the JA-and SA-related genes may play different roles in panicle leaf senescence from those of the BR-related genes. 

It has been well known that plant hormone signals can be transmitted to the nucleus by a series of signal transduction components to activate gene expression and regulate plant growth and development. Transcription factors are proteins that bind to a specific DNA sequence to control the transcription of specific genes. It has been shown that NAC, WRKY, and MYB transcription factors are central players in modulating transcriptional changes under hormonal control [16,17]. Apart from the plant hormone component encoding genes and many other hormone responsive genes, we also identified many NAC, WRKY, and MYB encoding genes from the hormone-related GO-term, such as WRKY51, WRKY70, transcription factor MYB6, MYB44, MYB108, NAC83, and NAC2 encoding genes. These transcription factors may play a crucial role in the LT-induced panicle leaf senescence. Future work should be carried out to examine the function of these central players.

To confirm our RNA-Seq data, we selected 10 plant hormone-related DEGs and determined their relative expression using qRT-PCR. The results showed that their expression trends as determined by qRT-PCR were similar to those revealed by RNA-Seq, and a significantly positive correlation between the obtained data from qRT-PCR and RNA-Seq was found, confirming the reliability of our RNA-Seq data. Our studies provided potential genes for the future molecular breeding of new cultivars that can induce panicle leaf senescence and reduce floral abortion under warm climates.

## 5. Conclusions

We have constructed eight RNA-Seq libraries of the developing and senescing panicle leaves, generated 4.78–8.99 × 10^6^ clean reads for each library, compared digital expression of the genes, identified 6477 upregulated DEGs (from developing leaves to senescing leaves), and 6318 downregulated DEGs. We also identified 158 ABA-, 68 ethylene-, 107 IAA-, 27 GA-, 68 CTK-, 37 SA-, and 23 BR-related DEGs that might be involved in panicle leaf senescence. Confirmation of our RNA-Seq data by qRT-PCR analysis suggested that our RNA-Seq data is reliable. Our studies provided potential genes for the future molecular breeding of new cultivars that can induce panicle leaf senescence and reduce floral abortion under warm climates.

## Figures and Tables

**Table 1:** Data quality and alignment analysis.

Sample	Raw Reads	Clean Reads	Clean Reads/Raw Reads (%)	Aligned Reads	Alignment Rate
DLE 1	6,521,358	6,395,241	98.07	3,825,633	59.82%
DLE 2	5,658,367	5,514,047	97.45	3,398,858	61.64%
DLL 1	7,370,305	6,518,556	88.44	3,761,206	57.70%
DLL 2	6,533,923	6,219,090	95.18	3,853,348	61.96%
SLE 1	9,081,562	8,998,048	99.08	5,485,210	60.96%
SLE 2	7,620,278	7,560,087	99.21	4,843,747	64.07%
SLL 1	4,825,239	4,781,295	99.09	3,219,724	67.34%
SLL 2	6,004,061	5,868,244	97.74	3,679,388	62.70%

DLE, developing leaves at an early stage. DLL, developing leaves at a later stage. SLE, senescing leaves at an early stage. SLL, senescing leaves at a later stage.

**Table 2:** Number of the differentially expressed genes.

Sample 1	Sample 2	Total	Up (Sample 1 to 2)	Down (Sample 1 to 2)
DLE	DLL	127	30	97
SLE	SLL	162	108	54
DLE	SLE	6662	3295	3367
DLL	SLL	6133	3182	2951

## Supplementary Materials

The following are available online at http://www.mdpi.com/2073-4425/10/2/111/s1, Table S1: Primer sequences of the reference and candidate genes for qRT-PCR, Figure S1: Correlation analysis of the sequencing samples, Figure S2: Gene ontology analysis of differentially expressed genes in the panicle leaves, Figure S3: Heat map diagram showing the JA-related differentially expressed genes (DEGs) between senescing and developing panicle leaves, Figure S4: Heat map diagram showing the SA-related differentially expressed genes (DEGs) between senescing and developing panicle leaves, Figure S5: Heat map diagram showing the BR-related differentially expressed genes (DEGs) between senescing and developing panicle leaves.
