# Supplementary material for: Identification of Genes Involved in Low Temperature-Induced Senescence of Panicle Leaf in Litchi chinensis
Source: Genes (Basel). 2019 Feb 1;10(2):111. doi: 10.3390/genes10020111 (PMC6409916; doi:10.3390/genes10020111)
Supplement: Supplementary file 1 [file genes-10-00111-s001.pdf]

Table S1. Primer sequences of the reference and candidate genes for qRT-PCR.

Homology gene	gene ID	Sequence F（5′→3′）	Sequence R（5′→3′）
Actin		AGTTTGGTTGATGTGGGAGAC	TGGCTGAACCCGAGATGAT
AGP31	Litchi_GLEAN_10038709	AAAAGCCCTAGCCCATACCGAC	GCTGACTCCACCATGCAAATCA
AHP	Litchi_GLEAN_10002497	GGTTTTGGATGGACAGTTTTTACA	TCTTTGTGCCCCTATGCTTGAG
IAA27	Litchi_GLEAN_10035463	TGTTCTCTCCTGCTGATACCAATG	GGCACCGTCCATACTGACTTTG
LHY	Litchi_GLEAN_10002826	GCGGGAGCGATGGACAGAGG	TTGGGGCGTGGTGGTGGAAT
NAC002-1	Litchi_GLEAN_10035859	TGGAAGCGGGTACTGGAAGG	AAGAGGCAGACGCAGAGCGA
NAC002-2	Litchi_GLEAN_10030627	TAAGGCTGGATGATTGGGTGC	TAGGATTTGGTGGCATGGCA
PEX5	Litchi_GLEAN_10007023	CACCAGAATCAAATCCCACCTC	GCCTTCAGACAATCCAAGCAA
RAP2-3	Litchi_GLEAN_10034724	ACTGCTGAAGAAGCCGCCAA	AGCCCCATACCCTCCAAACT
WRKY23	Litchi_GLEAN_10034637	TGGAATCTCCTGAGTTGTTGAATC	CGAATCTCGGCTCTCTCTGTCT
WRKY70	Litchi_GLEAN_10031581	TAAGGGGAGGAGAGGTTGTTACA	TGTGGGTTGTCTTCCATTTTTTG
VTC2	Litchi_GLEAN_10017404	CTCCCACCAAGAAGATAACCAC	TCCACAATCAGAAATGAGCACA
MYB32	Litchi_GLEAN_10038304	ACAGCCCTCGCTTAGATC	AGCCTGAAGTGGGTAGTG

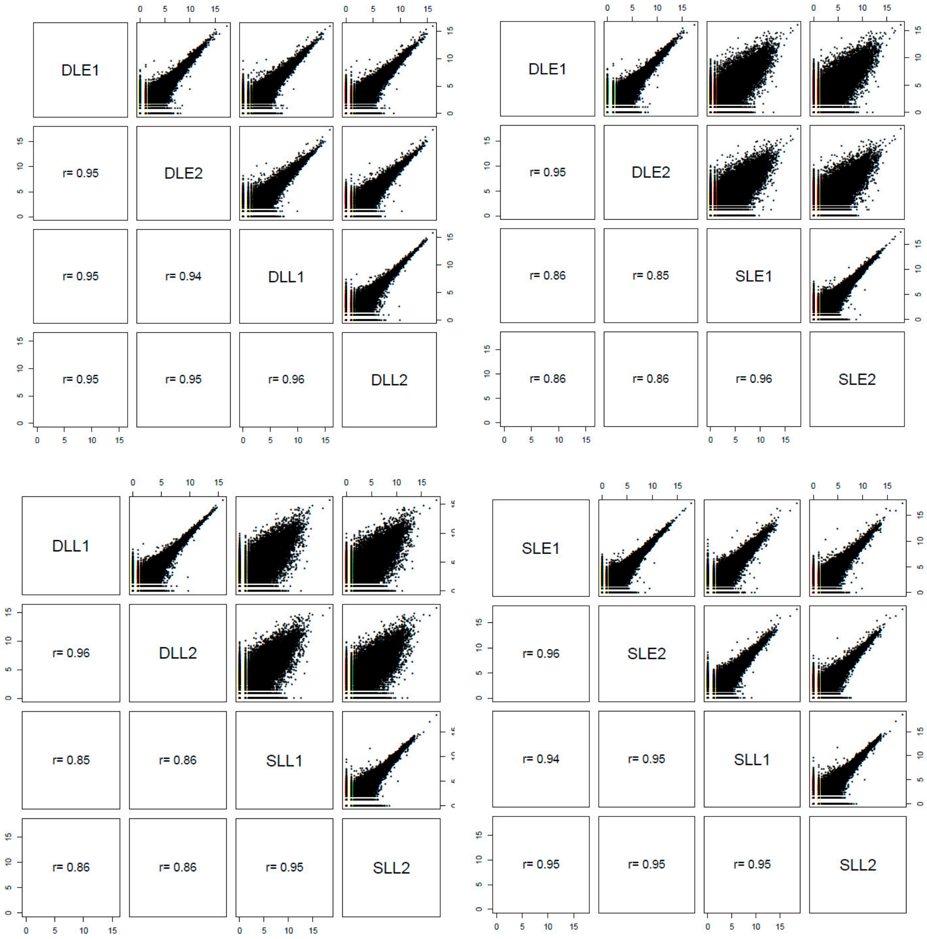

Figure S1. Correlation analysis of the sequencing samples

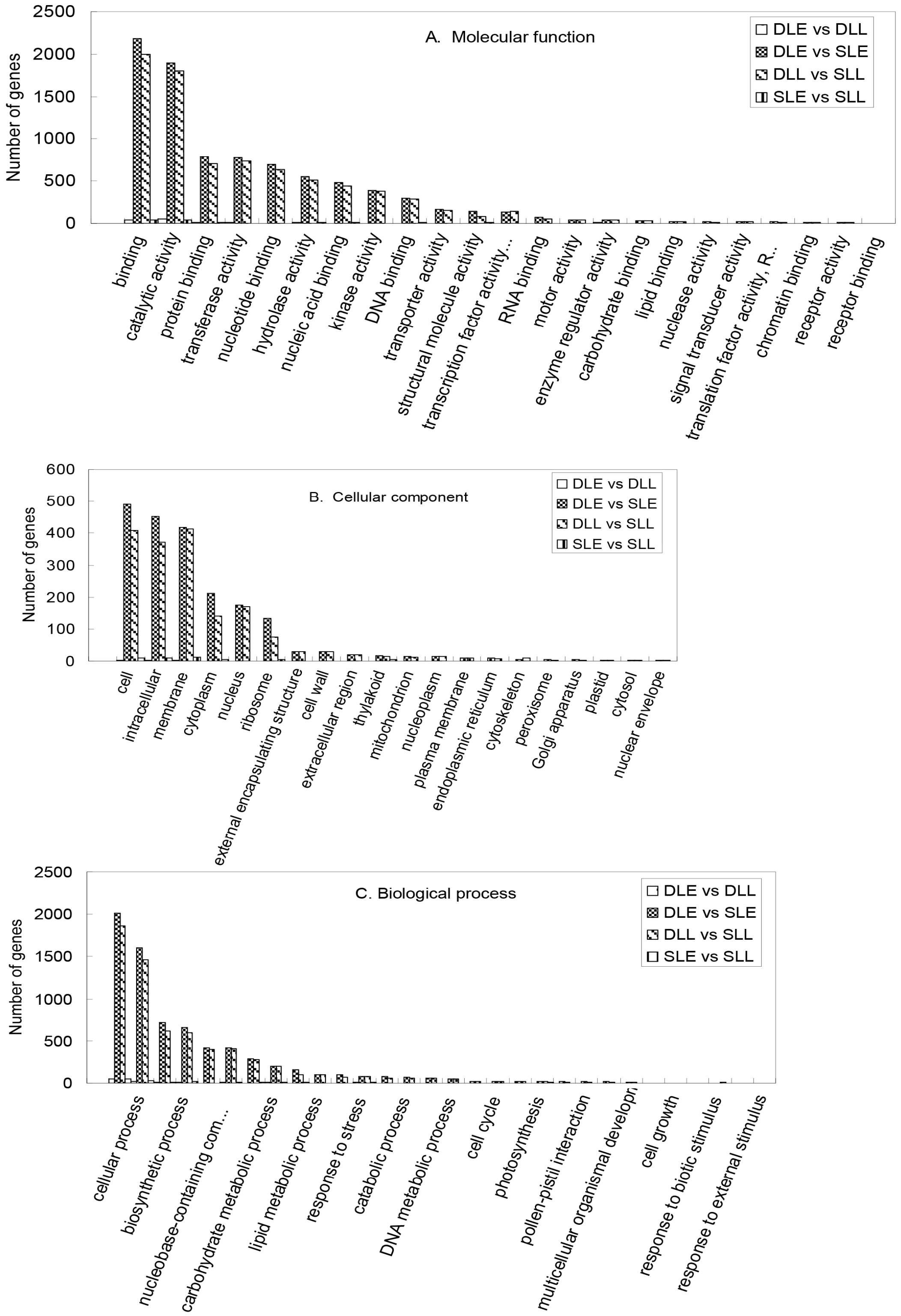

Figure S2. Gene ontology analysis of differentially expressed genes in the panicle leaves

A: molecular function; B: cellular component; C: biological process.

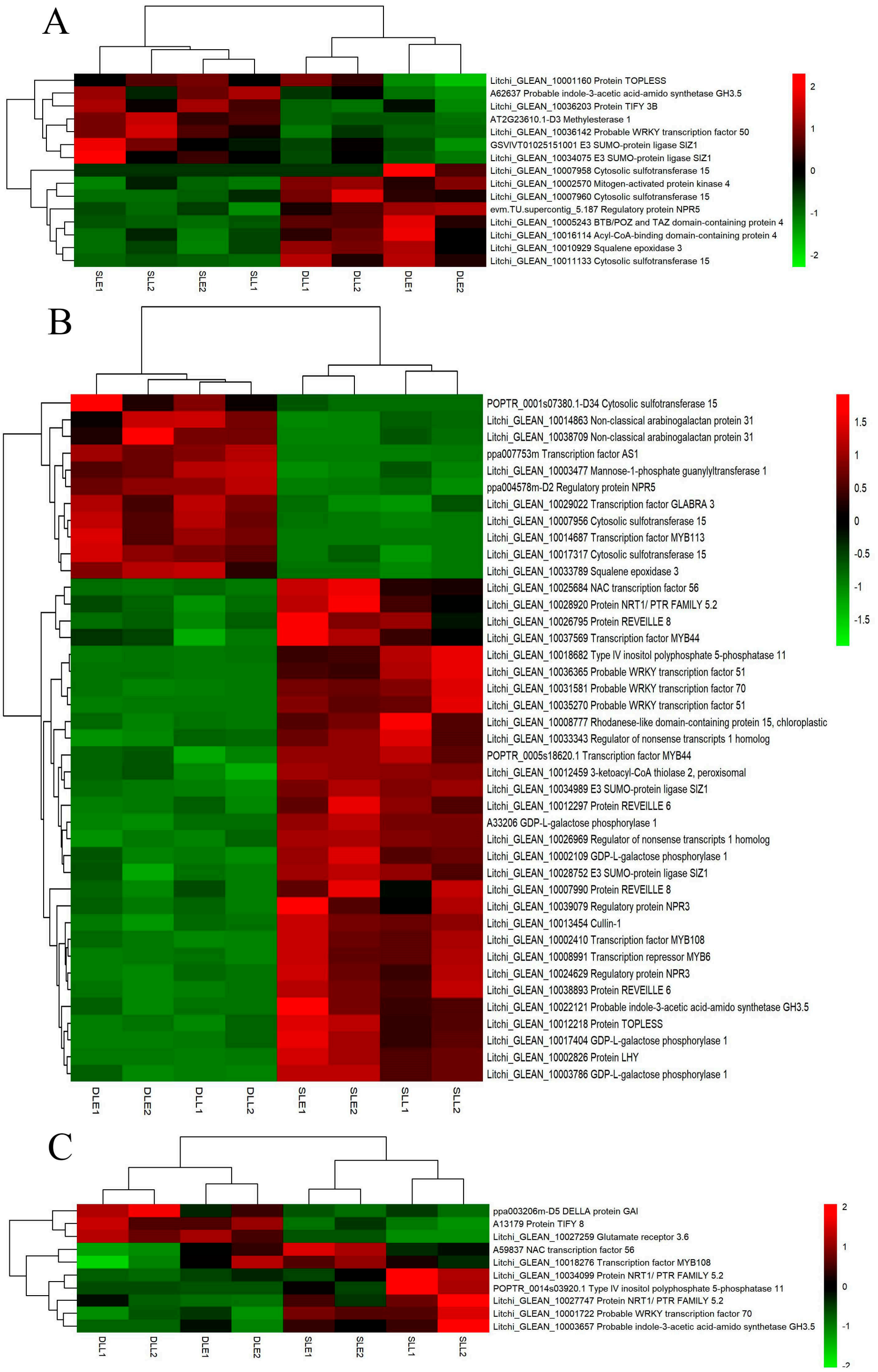

Figure S3. Heat map diagram showing the JA related differentially expressed genes (DEGs) between senescing and developing panicle leaves. A, the unique DEGs expressed at an early stage. B, the DEGs simultaneously expressed at early and later stages. C, the unique DEGs expressed at a later stage. DLE, developing leaves at the early stage. DLL, developing leaves at the later stage. SLE, senescing leaves at the early stage. SLL, senescing leaves at the later stage.

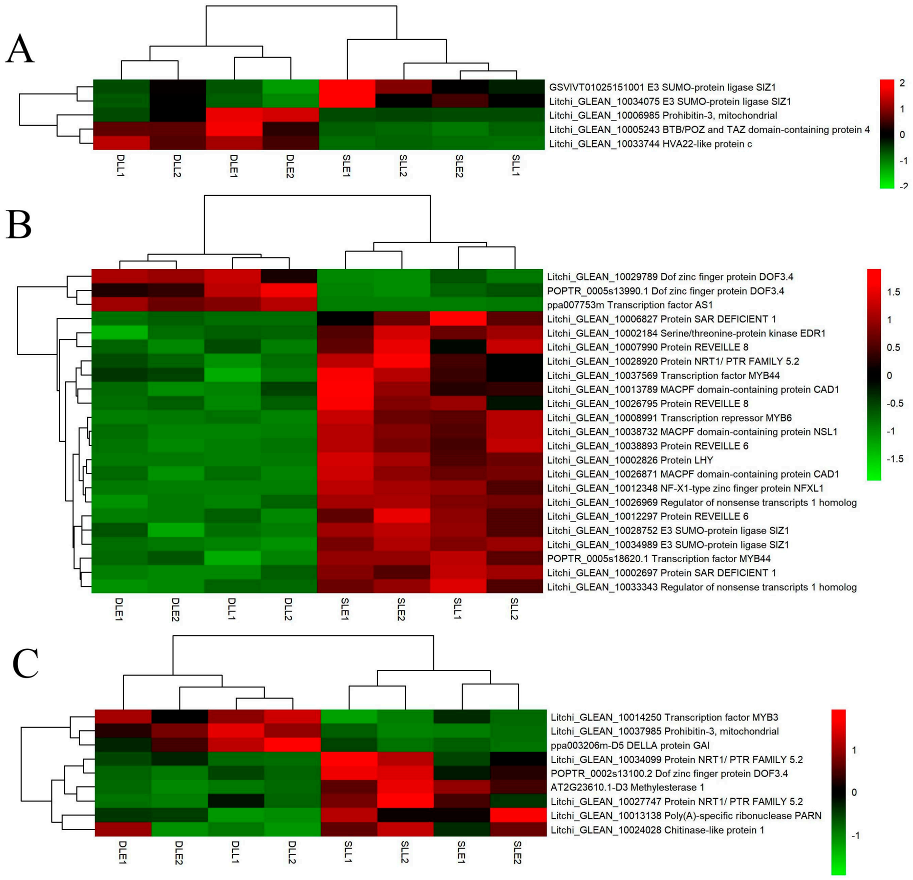

Figure S4. Heat map diagram showing the SA related differentially expressed genes (DEGs) between senescing and developing panicle leaves. A, the unique DEGs expressed at an early stage. B, the DEGs simultaneously expressed at early and later stages. C, the unique DEGs expressed at a later stage. DLE, developing leaves at the early stage. DLL, developing leaves at the later stage. SLE, senescing leaves at the early stage. SLL, senescing leaves at the later stage.

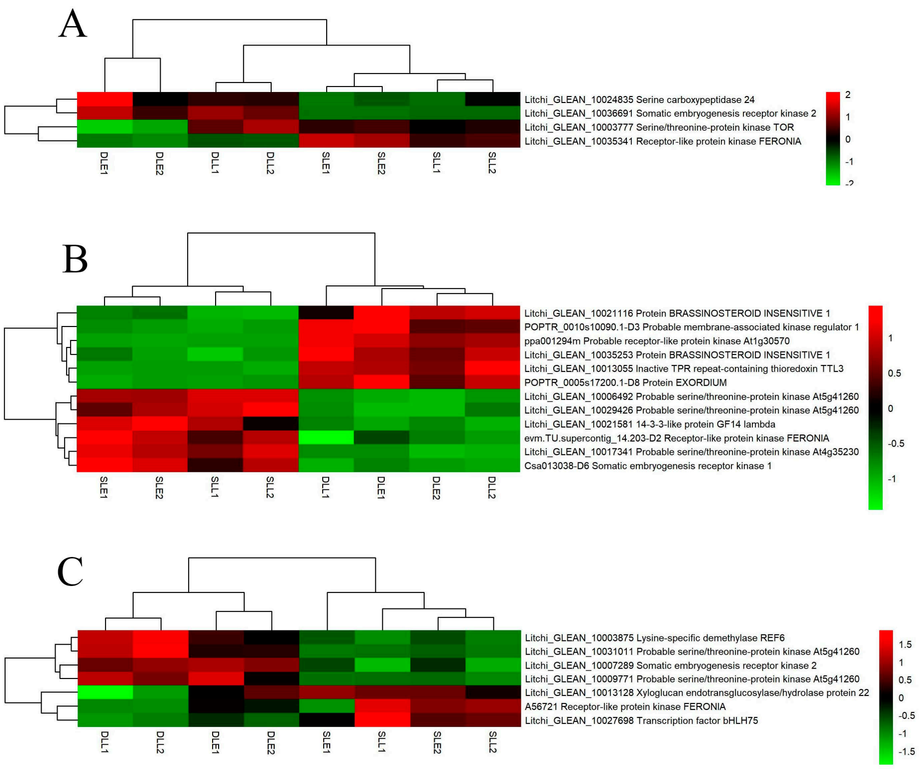

Figure S5. Heat map diagram showing the BR related differentially expressed genes (DEGs) between senescing and developing panicle leaves. A, the unique DEGs expressed at an early stage. B, the DEGs simultaneously expressed at early and later stages. C, the unique DEGs expressed at a later stage. DLE, developing leaves at the early stage. DLL, developing leaves at the later stage. SLE, senescing leaves at the early stage. SLL, senescing leaves at the later stage.
